# Staatsprogramme gegen die Corona-Krise — eine Option für den Klimaschutz?

**DOI:** 10.1007/s10273-020-2697-0

**Published:** 2020-07-18

**Authors:** Erik Gawel, Paul Lehmann

**Affiliations:** Department Ökonomie, Helmholtz-Zentrum für Umweltforschung, Permoserstr. 15, 04318 Leipzig, Deutschland

**Keywords:** D62, D72, H22, H23, Q48, Q55

## Abstract

Die Corona-Pandemie, insbesondere aber auch die Maßnahmen zu ihrer Eindämmung, führen aktuell zu einer schweren volkswirtschaftlichen Krise. Um diese zu bewältigen, wird von vielen Seiten staatliche Hilfe und Unterstützung erwartet. Zahlreiche Rettungs- und Ausgabenprogramme wurden beschlossen oder sind auf dem Weg. Eine wichtige Frage lautet dabei, in welcher Form Klima- und Umweltschutz bei der Ausgestaltung dieser Programme mitzudenken sind.

## Abstract (en)

Public measures to combat the coronavirus pandemic have led to a severe economic crisis. In order to cope with this crisis, many expect strong state intervention. Governments across the world have pledged billions of euros for extensive recovery programs. But how ‘green’ should these recovery programs be? This article evaluates Germany’s initial policy proposals and decisions.

Nach Beginn der Corona-Pandemie zeigten sich zunächst altbekannte politische Muster: In Krisenzeiten schlägt immer auch die Stunde der Interessenvertreter (Konrad und Thum, 2020). Wo in großer Eile riesige Summen öffentlicher Mittel bewegt werden, sind die Begehrlichkeiten naturgemäß groß. Die Chance, öffentliche Mittel im eigenen Sinne verwendet zu sehen, ruft Interessenvertreter aller Couleur auf den Plan, vorzugsweise mit alten Wunschlisten. Auf der einen Seite geriet dabei der Klimaschutz als „Kostenfaktor" unter Druck. So wurden aus CDU, FDP und vom Deutschen Industrie- und Handelskammertag Forderungen laut, zum jetzigen Zeitpunkt auf klimapolitische „Sonderbelastungen" – etwa die geplante Einführung eines $CO_2$-Preises für den Gebäude- und Verkehrssektor – zu verzichten, bis die Corona-Pandemie bewältigt sei. Die Automobilindustrie forderte, dass die auf EU-Ebene geplanten Verschärfungen der $CO_2$-Flottengrenzwerte verschoben werden sollten (Bauchmüller und Weiß, 2020; Götze, 2020; Kirchner 2020).

**Prof. Dr. Erik Gawel** leitet das Department Ökonomie am Helmholtz-Zentrum für Umweltforschung (UFZ) in Leipzig, ist Universitätsprofessor für institutionenökonomische Umweltforschung und Direktor des Instituts für Infrastruktur und Ressourcenmanagement der Universität Leipzig.

**Jun.-Prof. Dr. Paul Lehmann** lehrt Umwelt- und Energieökonomik, leitet die BMBF-Nachwuchsforschungsgruppe MultiplEE an der Universität Leipzig und ist wissenschaftlicher Mitarbeiter am UFZ.

Umgekehrt fordern andere, dass die Staatsgelder prioritär für Maßnahmen ausgegeben werden sollten, die auch dem Klimaschutz nützen. Frans Timmermans (Timmermans und Piccard, 2020), EU-Kommissar für Klimaschutz, und Dirk Messner (2020), Chef des Umweltbundesamts, mahnten, den „Kampf gegen das Coronavirus mit dem gegen Klimawandel und Umweltkrisen" zu verbinden. Umweltministerin Svenja Schulze (o. V., 2020) und Fatih Birol (2020), Chef der Internationalen Energieagentur, betonten, dass der Ausbau sauberer Energie Herzstück eines Konjunkturprogramms sein müsse. Die Linke forderte gar den Ausschluss von „Klimasündern" bei Rettungsgeldern und Konjunkturhilfen (Schultz, 2020).

In diesem polit-ökonomischen Wettbewerb darf jedoch nicht aus dem Blick geraten: Öffentliche Mittel sind weiterhin knapp und müssen planvoll eingesetzt werden. Werden jetzt die Subventionsschleusen unkontrolliert geöffnet, droht ein Ausgaben-Exzess, der künftige finanzpolitische Spielräume stark beschränkt. Auch grüne Subventionen können dann schnell Teil des Problems anstelle einer Lösung werden. Entscheidend ist es daher, zu Beginn jeder Diskussion um grüne Rettungs- und Ausgabenprogramme die Kriterien für die Auswahl von Maßnahmen transparent aufzuzeigen.

## Kriterien für grüne Subventionen in Krisenzeiten

Grüne Subventionen müssen zunächst stabilisierungspolitisch Sinn ergeben – sonst können sie im Wettstreit um die öffentlichen Mittel mit anderen wichtigen politischen Handlungsfeldern (Gesundheitssystem, Digitalisierung usw.) ohnehin nicht bestehen. Die Corona-Krise unterscheidet sich dabei deutlich von klassischen Wirtschaftskrisen, in denen typischerweise Markt-Eigendynamiken zu wirtschaftlichen Einbrüchen und Nachfrageschwächen führen. Ursachen sind jetzt vielmehr die aktuellen staatlichen Eingriffe zur Bekämpfung der Pandemie sowie die große Unsicherheit bezüglich ihres weiteren Fortgangs. Die Angebotskrise entsteht insbesondere durch den Zusammenbruch internationaler Lieferketten und Investitionszurückhaltung. Die Nachfragekrise wird durch Zwangs- und Angstsparen auf

Konsumentenseite verursacht (Felbermayr und Kooths, 2020; Feld et al., 2020). Mit staatlichen Ausgaben müssten daher gezielt diese Ursachen adressiert werden. Dabei ist zu beachten, dass sich manche Problemlagen auch ohne staatliches Zutun wieder auflösen könnten. So ist etwa zu erwarten, dass globale Lieferketten wieder aufgenommen und Käufe langlebiger Konsumgüter, wie z. B. Autos, zumindest teilweise nachgeholt werden.

Darüber hinaus müssen grüne Subventionen auch klimapolitisch sinnvoll sein. Dafür sollten sie insbesondere auf staatliche Maßnahmen fokussieren, die auch ohne die Corona-Krise volkswirtschaftlich sinnvoll gewesen wären. Dazu gehören insbesondere Maßnahmen, die Marktversagen (z. B. unzureichende Infrastrukturbereitstellung, Barrieren bei Technologieentwicklung und -diffusion) korrigieren, soweit diese durch bereits bestehende Instrumente wie etwa den europäischen $CO_2$-Emissionshandel noch nicht effizient behoben werden. Zusätzlich sollte das Hauptaugenmerk auf Maßnahmen liegen, die klimapolitisch Priorität haben und für die bereits reife Konzepte vorliegen, die jetzt schnell umgesetzt werden können. Es kann daher nicht darum gehen, jede grüne Technologie staatlich zu subventionieren. Entlang dieser Kriterien müssen die ersten Beschlüsse für Rettungs- und Ausgabenprogramme bewertet werden. Dabei müssen auch aktuelle politische Entscheidungen jenseits des Corona-Krisenmanagements – etwa die Beschlüsse zum Klimapaket und zur Reform des Erneuerbare-Energien-Gesetzes (EEG) – mitgedacht werden.

## Rettungsprogramme nach Betroffenheit und Bedürftigkeit ausrichten

Kurzfristige Rettungsprogramme müssen Menschen und Unternehmen rasch helfen, die unmittelbaren Folgen der Krise zu bewältigen. Die Vergabe der Mittel muss dabei nach zwei Kriterien erfolgen: Erstens müssen Einkommens- und Umsatzverluste unmittelbar durch die Corona-Krise verursacht sein, etwa aufgrund eines Nachfrageeinbruchs infolge staatlicher Zwangsmaßnahmen (Betroffenheit). Zweitens müssen diese Einbußen zu einer existenziellen Notlage geführt haben, die aus eigener Kraft nicht zu bewältigen ist (Bedürftigkeit). Einkommenseinbußen oder akute Nachfrageeinbrüche allein begründen also noch keinen Anspruch auf Rettungsgelder. Denn möglicherweise können aktuelle Verluste zumindest teilweise auch durch Rücklagen, kurzfristige Kredite oder Nachfrage zu einem späteren Zeitpunkt (wie bei vielen langfristigen Konsumgütern) kompensiert werden. Die Zielgenauigkeit vieler Ausgabevorschläge wird daher zu Recht kritisch betrachtet.

Schon dies verdeutlicht, dass es zur Bewältigung der Corona-Krise wenig zielführend wäre, Umweltgesetze außer Kraft zu setzen. Von diesem Ansatz profitierten Unternehmen am meisten, welche die Umwelt am stärksten verschmutzen. Die resultierende Entlastung stünde in keinem Zusammenhang zur tatsächlichen Betroffenheit und Bedürftigkeit. Dass sich derartige Stimmen bislang nicht haben durchsetzen können, ist als klimapolitischer (Teil-)Erfolg zu bewerten.

## Rettungspakete: klimapolitische Auflagen prüfen

Umgekehrt führen aber auch Forderungen zu weit, jetzt nur Unternehmen zu retten, die keine „Klimasünder“ sind. Ein solcher Strukturwandel mit der Brechstange wäre unverhältnismäßig. Niemand käme auf die Idee, die geplante Umstrukturierung einer Schiffsbesatzung dadurch zu lösen, dass man im Falle des Kenterns einigen gar keinen Rettungsring mehr zuwirft. Zudem wäre völlig unklar, nach welchen Kriterien sinnvollerweise zwischen „Klimasündern“ und „Klimafreunden“ unterschieden werden könnte. Die dazu ins Spiel gebrachte „Verpflichtung zu Klimaneutralität“ eignet sich jedenfalls kaum: Auch der Ölriese BP hat sich dies jedenfalls deklaratorisch schon auf die Fahnen geschrieben (Leitel, 2020).

Zudem sollten unternehmensspezifische Klimaschutzauflagen für Fördermaßnahmen kritisch geprüft werden. Diskutiert wurden diese insbesondere im Zusammenhang mit der Rettung der Lufthansa. Ende Mai 2020 hatte sich die Bundesregierung auf eine finanzielle Unterstützung der Lufthansa in Höhe von 9 Mrd. Euro durch eine 20 %ige Unternehmensbeteiligung in Form einer stillen Einlage sowie Staatskredite verständigt. Im Gegenzug verpflichtete sich die Lufthansa lediglich deklaratorisch zur Verfolgung von Nachhaltigkeitszielen einschließlich einer Erneuerung der Flotte. Viele Umweltschutzorganisationen hatten härtere Auflagen gefordert, etwa dass sich die Lufthansa zum Einsatz sauberer Kraftstoffe oder Verzicht auf Inlandsflüge nachprüfbar verpflichte (Schwarz, 2020). Die klimapolitische Wirkung solcher unternehmensspezifischer Auflagen wäre jedoch unsicher – etwa wenn Inlandsflüge durch Wettbewerber übernommen oder Emissionen im EU-Emissionshandel lediglich verlagert würden. Klimapolitisch zielführender wäre es daher, Fördermaßnahmen durch sektorübergreifende regulatorische Maßnahmen zu begleiten, etwa durch konsequente Besteuerung bestimmter Inlandsflüge oder die Förderung regenerativer Kraftstoffe. Stille Unternehmensbeteiligungen ohne Mitspracherecht und vage Absichtserklärungen verdünnen jedenfalls die Mittelzuweisung zu einem auch beihilferechtlich relevanten, zweckfreien Transfer. Die Zustimmung der EU-Kommission steht noch aus.

## Grüne Elemente im Konjunktur- und Zukunftspaket

Nach den ersten Rettungsprogrammen hat der Koalitionsausschuss Anfang Juni 2020 ein Ausgabenpaket mit einem Umfang von ca. 130 Mrd. Euro beschlossen (BMF, 2020; vgl. Abbildung 1). Dieses umfasst zum einen ein „Konjunk-

Abbildung 1

**Konjunktur- und Krisenbewältigungspaket und Zukunftspaket mit klimapolitisch relevanten Maßnahmen**

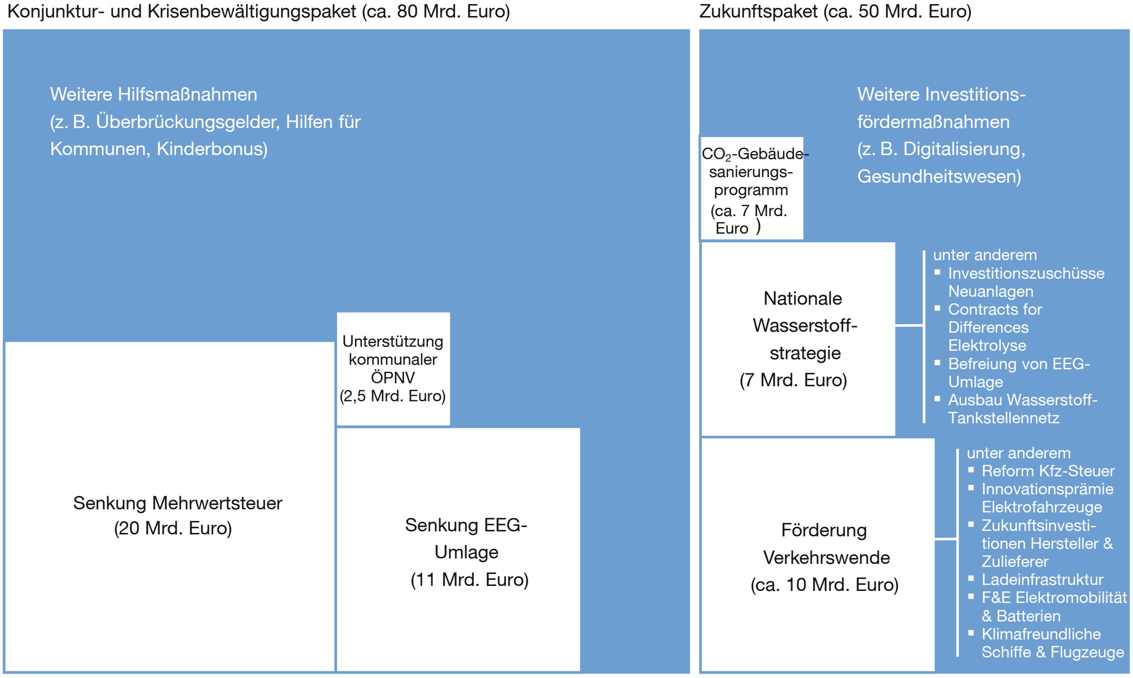

Quelle: eigene Abbildung.

tur- und Krisenbewältigungspaket“ mit weiteren Finanzhilfen sowie Maßnahmen zur kurzfristigen Stimulierung des Konsums. Wichtige Posten sind insbesondere die geplanten (teils befristeten) Senkungen von Umsatzsteuer und EEG-Umlage. Außerdem wurde ein Zukunftspaket beschlossen, das insbesondere Investitionen in Klimaschutztechnologien, Digitalisierung und das Gesundheitssystem fördern soll. Einige der beschlossenen Maßnahmen weisen also Bezüge zur Klima- und Energiepolitik auf. Sie ergänzen damit auch zeitgleich getroffene Beschlüsse etwa zum $CO_2$-Emissionshandel für den Verkehrs- und Gebäudesektor, zu einer $CO_2$-orientierten Kfz-Steuerreform oder zur Beschleunigung des Ausbaus von Windenergie und Photovoltaik.

Grundsätzlich ist es sinnvoll, Klimaschutz bei der Ausgestaltung des Konjunkturpakets mitzudenken. Die gegenwärtige Debatte über große staatliche Ausgabenprogramme schafft nicht nur ein beispielloses Gelegenheitsfenster für strukturellen Wandel. Die Hilfe durch die Allgemeinheit begründet auch eine besondere Gemeinwohl-Verpflichtung der Empfänger. Die politischen Gestaltungsspielräume sollten daher aktiv genutzt werden, um die notwendige Transformation von Wirtschaft und Gesellschaft hin zu mehr Nachhaltigkeit gezielt voranzubringen. Förderte der Staat jetzt mit Milliarden neue Investitionen in althergebrachte, $CO_2$-intensive Produktionsweisen und Technologien, würde der Weg hin zu Klimaneutralität zusätzlich erschwert. Insofern ist es zwar sinnvoll, Ausgabenprogramme auch nach klimapolitischen Kriterien zu gestalten – wie es gerade viele Akteure fordern (z. B. Agora Energiewende und Agora Verkehrswende, 2020; Bach et al., 2020; Dullien et al., 2020; Öko-Institut, 2020; UBA, 2020). Diese dürfen aber nicht beliebig sein. Eine kritische Prüfung der Vorschläge ist daher wichtig.

## Kein Subventionswettlauf mit der Gießkanne!

Grüne Konjunkturprogramme müssen mehr sein als grüne Subventionen. Wichtig ist der Abbau von unnötigen Investitionshemmnissen. Das gilt etwa für den Ausbau der erneuerbaren Energien. Bereits Mitte Mai 2020 war der Beschluss gefallen, den Ausbaudeckel für die Photovoltaik abzuschaffen und keine pauschalen Abstandsregelungen für die Windenergie an Land einzuführen. Diese Beschlüsse werden im Zukunftspaket bekräftigt. Nun muss es darum gehen, den Worten Taten folgen zu lassen und die notwendige, aber immer wieder verschobene EEG-Novellierung zügig durchzuführen.

Darüber hinaus gilt: Volkswirtschaftlich zielführend ist ein grünes Ausgabenprogramm nur, wenn gleichzeitig auch $CO_2$-intensives Wirtschaften durch einen $CO_2$-Preis und den Abbau umweltschädlicher Subventionen deutlich verteuert wird. Die Investitionsrichtung in der wirtschaftlichen Erholungsphase muss glasklar sein. Zudem würde so ein willkommener Beitrag zur Gegenfinanzierung der neuen Staatsausgaben geleistet. Interessanterweise fand dieser Punkt nur in wenigen Vorschlägen für grüne Konjunkturpakete Beachtung

(Ausnahme z.B. Bach et al., 2020). Ein entsprechendes Bekenntnis sucht man auch im Beschluss zum Zukunftspaket vergeblich. Dieses ist als reines Ausgabenprogramm angelegt. Entscheidend wird es daher sein, dass zeitlich parallele Beschlüsse zur Änderung des Brennstoff-Emissionshandelsgesetzes (BEHG) – insbesondere die geplante Erhöhung des $CO_2$-Festpreises von 10 auf 25 Euro/t $CO_2$ – zeitnah umgesetzt werden. Zwar kann es als Erfolg gewertet werden, dass die Einführung während der Corona-Krise trotz politischen Drucks mancher Interessengruppen nicht verschoben wurde. Wann und wie die Umsetzung erfolgt, ist aber weiter unklar – zumal rechtliches Ungemach droht, weil viele Juristen das BEHG in seiner jetzigen Form für verfassungswidrig halten (z.B. Kortländer 2020; Vollmer, 2020). Und selbst ein Preis von 25 Euro/t $CO_2$ läge wohl noch deutlich unter dem volkswirtschaftlich wünschenswerten $CO_2$-Preis. Die Expertenkommission zum Monitoring-Prozess „Energie der Zukunft" forderte jüngst 50 Euro/t $CO_2$ (Löschel et al., 2020).

Immerhin: Die bereits im Klimapaket versprochene $CO_2$-orientierte Reform der Kfz-Steuer wird im Zukunftspaket bekräftigt und liegt nunmehr als Kabinettsbeschluss vor. Dieser trägt freilich alle Zeichen von Symbolpolitik. 50 Cent der maximal 2 Euro Anhebung in der $CO_2$-Komponente sind allein durch Inflationsausgleich der seit Mitte 2009 unveränderten Steuersätze geboten (Gawel, 2011). Fortbestehende Hubraumkomponente und Freibetrag dämpfen weiter die Spreizung zwischen emissionsarmen und -intensiven Fahrzeugen, und die neue Tarif-Staffelung schont sorgsam die Kaufkraft im relevanten Massensegment. Die in letzter Minute noch hineinverhandelte Ermäßigung für besonders sparsame Verbrenner verringert zudem den Abstand zu echter Emissionsfreiheit bei Elektrofahrzeugen.

Eine wirksame Einpreisung der $CO_2$-Kosten kann jedoch durch kein noch so grünes Ausgabenprogramm ersetzt werden. Ohne einen flächendeckenden $CO_2$-Preis laufen grüne Subventionen Gefahr, zu verpuffen und durch die spätere Refinanzierungsnotwendigkeit dringend nötige Spielräume zu verkürzen. Soweit staatliche Nachhaltigkeitspolitik darüber hinaus im Rahmen der jetzt durch den Koalitionsausschuss beschlossenen Krisen- und Investitionsprogramme auch durch grüne Subventionen erfolgen soll, ist zu prüfen, ob diese den eingangs erwähnten stabilitäts- und klimapolitischen Kriterien gerecht werden.

### Mehrwertsteuer- und EEG-Umlagesenkung fragwürdig

Zentrale Säulen des „Konjunktur- und Krisenbewältigungspakets" sind eine auf sechs Monate begrenzte Senkung der Umsatzsteuer sowie die Bezuschussung der EEG-Umlage. Mit der Umsatzsteuersenkung gelang der Koalition ein Überraschungscoup. Im Gegensatz dazu war eine Senkung der EEG-Umlage praktisch von allen politischen Akteuren und auch führenden Ökonomen gefordert worden (z.B. Feld et al., 2020). Dabei ist sowohl der stabilitäts- als auch der klimapolitische Sinn derartiger pauschaler Vergünstigungen für Güter und Dienstleistungen zumindest fragwürdig.

Stabilitätspolitisch besteht die Idee darin, durch Preissenkungen den Konsum anzukurbeln. Es ist aber unklar, ob und in welchem Umfang die Kostensenkungen tatsächlich bei den Verbrauchern ankommen. Während Erhöhungen staatlich induzierter Kostenbestandteile von Unternehmen typischerweise im Preis weitergereicht werden, gilt das für entsprechende Senkungen nicht notwendigerweise (Peltzman, 2000). Stromverbraucher, die ihre Stromrechnung mit festen monatlichen Abschlägen bezahlen, profitierten von einer Umsatzsteuer- und EEG-Umlage-Senkung zudem in jedem Fall frühestens erst bei der nächsten Jahresabrechnung. Man mag einwenden, dass ohne Weiterreichung zumindest die Angebotsseite profitiert. Die für das Gastgewerbe schon vorgezogene befristete Absenkung auf den ermäßigten Satz zielte darauf ab, kann aber die dortigen Umsatzausfälle gar nicht adressieren. Das bedeutet aber, dass es im Wesentlichen von den Elastizitäten der Angebots- und Nachfragefunktionen in den jeweiligen Sektoren abhängt, welche Marktseite wie stark profitiert. Ob und wie stark die einzelnen Sektoren sowie die jeweiligen Marktseiten von der Krise betroffen sind, bleibt damit unberücksichtigt und zeigt eine wenig zielgenaue Wirtschaftspolitik. Und selbst wenn die Preissenkungen weitergereicht werden, bleibt unklar, ob derartige Maßnahmen tatsächlich den Konsum ankurbeln, zumal die Steuersenkung zwar für die öffentliche Hand Milliardenausfälle bedeutet, bei Gütern des täglichen Bedarfs aber kaum messbare Beträge bedeutet. Die Unsicherheit über den Fortgang der Pandemie bleibt. Viele Branchen (z.B. Tourismus, öffentlicher Verkehr, Veranstaltungsmanagement) befinden sich zudem weiter im Krisenmodus. Wahrscheinlich ist daher, dass Preissenkungen eher für „Angstsparen" als für zusätzlichen Konsum verwendet werden. Zudem drohen insbesondere bei langlebigen Gütern Mitnahme- und Vorzieheffekte ohne wirkliche Nachfragesteigerung. Zielgerichtete Stabilitätspolitik geht anders. Das ist insbesondere problematisch, weil derartig ungenaue Maßnahmen gleichzeitig mit hohen Einbußen für die öffentlichen Haushalte einhergehen. Wesentlich zielgerichteter wirken könnten daher direkte Transfers an Unternehmen und Menschen, z.B. in Form erleichterter Verlustvor- und -rückträge, als Verlängerungen des Kurzarbeitergeldes oder als Erstattung von Umsatzausfällen für Soloselbständige.

Außerdem werfen beide Maßnahmen auch klimapolitische Fragen auf. In Sektoren ohne zureichende $CO_2$-Bepreisung wird eine pauschale Umsatzsteuersenkung bewirken, dass suboptimal viele emissionsintensive Güter und Dienstleistungen nachgefragt werden. Das Zusammenspiel aus Umsatzsteuersenkung 2020 und Kfz-Steuererhöhung 2021 kann so ganz nebenbei zu einer impliziten „Kaufprämie" für

Sofort-Autokäufe führen, die umso größer ausfällt, je teurer und $CO_2$-trächtiger das Fahrzeug ist. Zur klimafreundlichen Umwälzung der Fahrzeugflotte trägt dies wenig bei. Auch der klimapolitische Sinn einer EEG-Umlage-Senkung erschließt sich nicht. Zwar kann von einer entsprechenden Senkung der Betriebskosten möglicherweise die Elektrifizierung von Verkehrs- und Wärmesektor profitieren. Die eigentlichen Hemmnisse dafür, die zumindest punktuell auch staatliche Subvention rechtfertigen würden, bestehen jedoch in der mangelnden Infrastruktur sowie hohen Investitionskosten. Dort wären staatliche Fördergelder in jedem Fall effizienter angelegt. Zudem muss grundsätzlich gefragt werden, ob Stromverbilligung der richtige Weg ist, um die Elektrifizierung voranzubringen. Da insbesondere erneuerbarer Strom ein knappes Gut ist, sind Stromsparanreize gerade auch bei der Transformation hin zu einer elektrifizierten Mobilität und Wärmeversorgung essenziell (Lehmann et al., 2020).

## Zukunftspaket: Richtung stimmt, Detailfragen bleiben

Dass im Zukunftspaket Schwerpunkte auf die Förderung von Verkehrswende, Wasserstoffwirtschaft und Gebäudesanierung gelegt werden, ist grundsätzlich nachvollziehbar. Hier besteht klimapolitisch großer Nachholbedarf und zum Teil auch Förderindikation. Zudem haben Akteure wie Agora Verkehrswende bereits konkrete Maßnahmenprogramme entwickelt (Lehmann et al., 2020). Der Teufel steckt dabei jedoch oft im Detail, wie das Beispiel der im Verkehrssektor geplanten Maßnahmen zeigt.

Bemerkenswert am verkehrspolitischen Maßnahmenpaket ist dabei zunächst, welche Maßnahme nicht darin vorkommt: eine universelle Abwrackprämie für Autos auch mit Verbrennungsmotor. Das ist insbesondere in Anbetracht des massiven Drucks der Automobilindustrie sowie der Bundesländer ein klimapolitischer Erfolg. Die Entscheidung ist auch stabilitätspolitisch vertretbar. Eine Abwrackprämie hätte zu einer Vorverlagung von Autokäufen geführt und ein umso tieferes Absatzloch im Folgejahr verursacht. Auch viele Ökonomen hatten sich daher dagegen ausgesprochen (z.B. Feld et al., 2020). Die befristete Umsatzsteuersenkung in Verbindung mit der Kfz-Steuererhöhung 2021 schafft jedoch trotzdem Anreize, jetzt ein Fahrzeug mit Verbrennungsmotor anzuschaffen. Eine explizite Kaufprämie (Innovationsprämie) soll es jedoch für Elektrofahrzeuge geben. In begrenztem Umfang können derartige Kaufprämien ökonomisch begründet sein (Budgetrestriktionen auf der Nachfrageseite, Barrieren bei der Technologiediffusion). Unklar ist aber, inwieweit von der jetzt beschlossenen Verdopplung des bereits vorhandenen staatlichen Zuschusses auf 6000 Euro tatsächlich noch ein zusätzlicher Impuls ausgehen kann. Zumal der Absatz von Elektrofahrzeugen bereits jetzt aufgrund von Lieferengpässen hakt. Kritisch zu bewerten ist zudem, dass die Option einer Förderung von Plug-in-Hybridfahrzeugen erwogen wird. Deren Klimanutzen ist zumindest unklar, wenn der elektrische Fahranteil nicht kontrolliert werden kann. Entscheidender für eine Antriebswende im Verkehrssektor wird jedoch sein, dass insbesondere die Ladeinfrastruktur ausgebaut wird und die Batterietechnologien weiterentwickelt werden. Beide Posten sind im Zukunftspaket vermerkt. Doch auch hier gilt: Entsprechende Beschlüsse waren bereits im Klimapaket verabschiedet worden (z.B. 1 Mio. Ladesäulen), harren bisher jedoch noch ihrer Umsetzung.

In ähnlicher Weise muss auch für die nationale Wasserstoffstrategie noch mal geprüft werden, inwieweit grüne Subventionen aufgrund von Marktversagen tatsächlich gerechtfertigt sind – oder aber nicht bereits ein wirksamer $CO_2$-Preis ausreichende Signale für den Ausbau einer Wasserstoffwirtschaft setzen würde. Eine zeitlich und vom Umfang her begrenzte Anschubförderung von Infrastruktur und Technologieentwicklung scheint ökonomisch geboten zu sein, die Sinnhaftigkeit einer pauschalen EEG-Umlage-Befreiung stünde jedoch z.B. auch hier infrage. Zudem sollen die angehefteten Milliardenbeträge scheinbar zunächst vor allem politische Symbolkraft entfalten. Eine genaue Prüfung des tatsächlich notwendigen Finanzierungsbedarfs steht wohl noch aus. Gleiches gilt für die geplante Aufstockung des Gebäudesanierungsprogramms.

Generell darf sich die Diskussion um (grüne) Rettungs- und Konjunkturprogramme aber nicht allein auf die Ausgabenseite beschränken. Völlig unterbelichtet ist gegenwärtig die Frage, woher all die Milliarden kommen sollen. Hier hat der Beschluss des Koalitionsausschusses eine Fehlstelle, die auch durch die geplante Kfz-Steuerreform und die Einführung es $CO_2$-Preises für Gebäude und Verkehr nicht angemessen geschlossen wird. Es sollte jedoch erwogen werden, die grünen Ausgabenprogramme zumindest teilweise klimapolitisch verursachergerecht durch einen spürbaren $CO_2$-Preis und die überfällige Abschaffung umweltschädlicher Subventionen aufzubringen (Bach et al., 2020; Gawel und Purkus, 2015). Diese Instrumente der Abgeltung bisher nicht eingepreister Umweltfolgen wären gerade kein „Sonderopfer", sondern Teil der Lösung – sowohl für das Einnahmenproblem als auch das Umsteuern von Produktion und Konsum in Richtung Zukunftsfähigkeit.

## Fazit: Stabilisierung, Klimaschutz und Finanzierung zusammendenken

Das durch die Corona-Krise weit geöffnete Transformationsfenster muss über die anstehenden Ausgabeprogramme aktiv für Nachhaltigkeitsakzente genutzt werden, ohne dabei ordnungspolitisch vom Kurs abzukommen. Klimapolitik darf sich nicht in grünen Subventionen erschöpfen. Gezielte Beseitigung von Investitionshemmnissen und spürbare $CO_2$-Preise sind ebenso wichtig. Der stabilisierungspolitische Teil der Ausgabeprogramme muss an den Spezifika der Krise ansetzen, insbesondere das Angst- und weitere Zwangssparen in bestimmten Branchen adressieren. Komplizierte Umsatzsteuergeschenke sind aufwendig und teuer, aber viel zu wenig effektiv. Und befristete Umsatzsteuergeschenke programmieren absehbar Prolongationsforderungen. Für eine Kfz-Kaufprämie über Umwege ist dies wohl die teuerste und klimapolitisch unergiebigste Variante. Staatliche Mittel an Umweltauflagen zu knüpfen, bleibt im Einzelfall schwierig, ersetzt in jedem Fall keine Gesamtstrategie. Es fehlt weiter eine klare, sektorübergreifende $CO_2$-Bepreisung, die nicht nur einen Teil-Beitrag zur Finanzierung der staatlichen Mehrausgaben leisten könnte, sondern zugleich klare Anreize für zukunftsfähiges Wirtschaften setzen würde.
